# Adherence to yoga and exercise interventions in a 6-month clinical trial

**DOI:** 10.1186/1472-6882-7-37

**Published:** 2007-11-09

**Authors:** KE Flegal, S Kishiyama, D Zajdel, M Haas, BS Oken

**Affiliations:** 1 Department of Neurology, Oregon Health & Science University, Portland, USA; 2 Department of Behavioral Neuroscience, Oregon Health & Science University, Portland, USA; 3 Center for Outcome Studies, Western States Chiropractic College, Portland, USA

## Abstract

**Background:**

To determine factors that predict adherence to a mind-body intervention in a randomized trial.

**Design:**

We analyzed adherence data from a 3-arm trial involving 135 generally healthy seniors 65–85 years of age randomized to a 6-month intervention consisting of: an Iyengar yoga class with home practice, an exercise class with home practice, or a wait-list control group. Outcome measures included cognitive function, mood, fatigue, anxiety, health-related quality of life, and physical measures. Adherence to the intervention was obtained by class attendance and biweekly home practice logs.

**Results:**

The drop-out rate was 13%. Among the completers of the two active interventions, average yoga class attendance was 77% and home practice occurred 64% of all days. Average exercise class attendance was 69% and home exercise occurred 54% of all days. There were no clear effects of adherence on the significant study outcomes (quality of life and physical measures). Class attendance was significantly correlated with baseline measures of depression, fatigue, and physical components of health-related quality of life. Significant differences in baseline measures were also found between study completers and drop-outs in the active interventions. Adherence was not related to age, gender, or education level.

**Conclusion:**

Healthy seniors have good attendance at classes with a physically active intervention. Home practice takes place over half of the time. Decreased adherence to a potentially beneficial intervention has the potential to decrease the effect of the intervention in a clinical trial because subjects who might sustain the greatest benefit will receive a lower dose of the intervention and subjects with higher adherence rates may be functioning closer to maximum ability before the intervention. Strategies to maximize adherence among subjects at greater risk for low adherence will be important for future trials, especially complementary treatments requiring greater effort than simple pill-taking.

## Background

Adherence is an important consideration in clinical trial design, especially concerning mind-body interventions. A meta-analysis of adherence to a range of medical interventions, not just drug therapy, found that adherence increases the likelihood of a good outcome by 26%[1] The effect of high adherence on better health outcomes has even been documented in the placebo arm of a double blind randomized trials [2-4]. Subject characteristics that determine adherence to drug treatment regimens and behavioral interventions have been found to include psychological states such as depression, anxiety, and psychological distress; cognitive-motivational factors such as health beliefs, intentions, and self-efficacy; somatic and cognitive appraisals such as perceived physical fitness and adverse side effects of a treatment; and health-oriented behaviors [5]. In a mind-body intervention, where greater effort than simple pill-taking is required, factors that predict adherence and strategies to maximize adherence deserve special attention. One reason is that adherence is lower with behavioral interventions than with pill-taking [6] and adherence may contribute to the effectiveness of the intervention. The other is that the relationship between adherence and health outcomes may itself be mediated through mind-body mechanisms, with potential roles for expectancy, self-efficacy, and interest in health promoting activities. These interactions, if not adequately controlled for, may potentially confound results of mind-body trials, especially since the trials are not blinded to the participants out of necessity. For example, if there is a large adherence effect on the outcomes, it may be necessary to stratify allocation to better match adherence in the groups. The present study investigates correlates of adherence to yoga and exercise interventions in a population of healthy seniors, contributing evidence for baseline measures including mood, physical functioning, general health, and fatigue impacting adherence rates and probability of study completion.

## Methods

The adherence data presented here were collected from a recently reported 3-arm clinical trial [7]. The study consisted of 135 generally healthy men and women 65–85 years of age randomized to a 6-month intervention consisting of: an Iyengar yoga class along with home practice, an exercise class along with home practice, or a wait-list control group given the option of enrolling in either of the active interventions after the wait-list period. As reported [7], the yoga intervention produced improvement in quality of life and physical measures but not on cognition or mood.

Subjects in the yoga intervention were taught beginning Iyengar hatha yoga poses in weekly 90-minute classes. Daily home practice was strongly encouraged, and subjects were given a booklet illustrating the specific poses to help with their independent practice [8]. Subjects in the exercise intervention engaged in aerobic walking on an outdoor track in weekly 60-minute classes. Daily home exercise in the same range of perceived exertion was encouraged at least 5 times per week. Subjects in the wait-list group participated in no active intervention. All subjects received monthly phone calls to assess for changes in health.

Baseline assessments of study outcome measures were performed before subjects were randomized and occurred 1 to 30 days before classes started. On the baseline visit, medical history was reviewed, demographic data were recorded, and the oral reading on the Wide Range Achievement Test, 3rd edition (WRAT-3) [9], was administered to assess equality of educational achievement in the three intervention groups. All outcome assessments were done at baseline and 6 months. There was also a 3-month visit primarily designed to encourage subjects' continued participation in the study

Outcome measures included cognitive function, mood as assessed by Center for Epidemiologic Studies Depression Scale (CESD-10) [10] and Profile of Mood States (POMS) [11], fatigue as assessed by Multidimensional Fatigue Inventory (MFI-20) [12], state and trait anxiety (STAI) [13], health-related quality of life (SF-36) [14], and physical measures (seated forward bend, one legged-standing). Adherence to the each of the active interventions was obtained by class attendance and biweekly home practice logs. In a similar manner to other published trials of physical activity with measures of adherence (e.g., Martin and Sinden [15]) the percent of days home practice occurred (frequency) and the average length of home practice sessions (duration) were calculated.

## Results

Subject demographics and adherence data are summarized in Table 1. 135 subjects were randomized and 118 completed the study by participating in the 6-month assessment, yielding a total drop-out rate of 13%. Attrition rates from the yoga, exercise, and wait-list groups were 14%, 19%, and 5%, respectively, not significantly different by chi-square. Within the two active interventions, adherence data were available from 36 of the 38 completers in the yoga condition and all 38 completers in the exercise condition. For the yoga group, average attendance at classes was 77% and home practice occurred on 64% of all days, while for the exercise group class attendance was 69% and home exercise occurred on 54% of all days (or 76% of the five days a week it was recommended). The adherence differences between the yoga and the exercise group did not reach statistical significance (for percent attendance, t = -1.94, p = 0.056 and for percent days practiced out of all days possible, t = -1.822, p = 0.073). Home practice sessions lasted an average of 38 minutes for the yoga group and 56 minutes for the exercise group (t = 3.8, p = .0003). The objective measure of class attendance was correlated with the self-report measures of adherence: number of days and length per day (Wilcoxon, *p*'s < 0.0005). Class attendance was selected for the primary analyses because it was the most objective measure.

**Table 1:** Subject demographics and adherence.

	Exercise (n = 47)	Yoga (n = 44)	Wait-List (n = 44)
Age	73.7 ± 5.1	71.5 ± 4.9	71.2 ± 4.4
Gender	79% female	70% female	75% female
Years of Education	14.8 ± 2.8	15.5 ± 2.2	15.3 ± 2.8
WRAT-3 Reading	49.0 ± 4.0	48.7 ± 4.3	49.0 ± 3.4
*Measures of Adherence:*			
Percent Classes Attended	69 % ± 19 %	77 % ± 20 %	-
Percent Days Home Practice	54 % ± 26 %	64 % ± 22 %	-
Average Daily Home Practice (min.)	56.1 ± 26.4	38.2 ± 10.4	-
Study completer vs. drop-out (% drop-out)	19%	14%	5%

Means ± SDs are reported

Adherence as measured by class attendance was significantly correlated with several baseline measures including mood, physical aspects of quality of life, and measures of fatigue (see Table 2). Higher baseline scores on self-rated measures of depression and fatigue were associated with lower class attendance during the 6 months of the intervention, while there was a positive relationship between adherence and self-rated measures of physical functioning, general health, vitality, and vigor at baseline.

**Table 2:** Correlations of adherence (Percent Classes Attended) and baseline variables among study completers.

		Exercise (n = 38)	Yoga (n = 36)	Both active interventions
	Age	-0.08	0.07	-0.07
	Gender	-0.20	0.12	-0.05
	Years of Education	-0.10	-0.29	-0.12
	WRAT-3 Reading	0.18	-0.09	0.01
	CES-D:10	-0.24	**-0.36**	**-0.27**

STAI				
	State Anxiety	-0.08	-0.33	-0.20
	Trait Anxiety	-0.01	**-0.41**	-0.23

SF-36				
	Physical Functioning	0.21	**0.52**	**0.38**
	Role-Physical	0.31	0.23	**0.27**
	Bodily Pain	0.22	0.20	0.21
	General Health	0.15	0.31	**0.23**
	Vitality	0.23	**0.38**	**0.32**
	Social Functioning	0.17	-0.04	0.09
	Role-Emotional	0.19	0.16	0.18
	Mental Health	0.17	0.13	0.13
	Physical Composite	0.25	**0.41**	**0.33**
	Mental Composite	0.15	0.09	0.13

POMS				
	Tension-Anxiety	0.12	**-0.42**	-0.16
	Depression-Dejection	-0.06	-0.15	-0.08
	Anger-Hostility	0.06	-0.10	-0.01
	Vigor	0.05	**0.36**	**0.25**
	Fatigue	-0.11	**-0.52**	**-0.34**
	Confusion	0.01	-0.23	-0.12
	Total Mood Disturbance	-0.01	**-0.36**	-0.20

MFI-20				
	General Fatigue	**-0.33**	**-0.56**	**-0.44**
	Physical Fatigue	-0.01	**-0.34**	-0.17
	Reduced Activity	-0.06	**-0.38**	-0.21
	Reduced Motivation	0.06	**-0.37**	-0.17
	Mental Fatigue	-0.09	-0.32	-0.19

Correlation coefficients in **bold **are significant at *p *< 0.05

Additionally, baseline measures of physical function and fatigue were significantly different between subjects who completed the study and those who dropped out (see Table 3). At baseline, those subjects who went on to complete the study scored higher on self-rated measures of physical functioning and general health, and lower on self-rated measures of trait anxiety and general fatigue, than those subjects who eventually dropped out. Of interest, completers had a higher mean baseline rating of bodily pain on the SF-36 questionnaire than did drop-outs.

**Table 3:** Active intervention completers and drop-outs compared on baseline variables.

		Completers (n = 76)	Drop-outs (n = 15)	*p*-value
	Age	72.4 ± 5.1	73.8 ± 5.1	0.33
	Gender	74% female	80% female	0.61
	Years of Education	15.0 ± 2.4	15.6 ± 3.3	0.48
	WRAT-3 Reading	48.9 ± 3.9	48.1 ± 6.6	0.63
	CES-D:10	4.9 ± 3.8	6.6 ± 4.4	0.14

STAI				
	State Anxiety	28.5 ± 8.6	33.0 ± 9.2	0.07
	Trait Anxiety	29.5 ± 7.7	34.4 ± 8.4	**0.03**

SF-36				
	Physical Functioning	83.5 ± 13.0	72.5 ± 14.1	**0.005**
	Role-Physical	78.6 ± 31.0	67.9 ± 34.6	0.24
	Bodily Pain	74.8 ± 19.3	61.1 ± 19.3	**0.02**
	General Health	81.0 ± 15.4	65.1 ± 13.7	**0.001**
	Vitality	69.2 ± 16.0	65.7 ± 12.4	0.44
	Social Functioning	91.9 ± 14.7	88.4 ± 18.0	0.43
	Role-Emotional	82.0 ± 29.0	83.3 ± 25.3	0.87
	Mental Health	84.1 ± 12.4	81.4 ± 12.4	0.45
	Physical Composite	49.4 ± 7.0	42.7 ± 6.9	**0.001**
	Mental Composite	54.7 ± 6.9	55.5 ± 6.3	0.69

POMS				
	Tension-Anxiety	0.8 ± 4.3	2.4 ± 4.5	0.19
	Depression-Dejection	4.3 ± 5.4	4.8 ± 4.4	0.77
	Anger-Hostility	3.7 ± 5.0	5.3 ± 5.9	0.31
	Vigor	20.4 ± 4.8	19.1 ± 5.1	0.37
	Fatigue	4.3 ± 3.8	5.6 ± 4.9	0.24
	Confusion	0.4 ± 3.2	0.6 ± 2.4	0.88
	Total Mood Disturbance	-6.9 ± 19.8	-0.4 ± 21.4	0.27

MFI-20				
	General Fatigue	8.5 ± 3.5	10.5 ± 2.7	**0.04**
	Physical Fatigue	7.8 ± 3.0	9.2 ± 3.1	0.11
	Reduced Activity	8.1 ± 3.5	9.5 ± 3.1	0.14
	Reduced Motivation	6.9 ± 2.7	8.1 ± 3.9	0.12
	Mental Fatigue	7.7 ± 3.5	8.8 ± 2.7	0.24

Means ± SDs are reported

Operationalized as either class attendance or study completion, adherence was not related to age, gender, or education level. There was no obvious effect of adherence on the significant outcomes from the intervention (quality of life and physical measures), defining class attendance continuously or as a threshold (adherers greater than 60% class attendance).

A limited number of subjects (n = 29) also completed a five-factor personality inventory, the NEO PI-R [16]. No significant relationships between adherence and personality were found (Pearson's correlation, all p values greater than 0.25), although the small sample size constrained the power to determine an effect.

## Discussion

Consistent with other reports of behavioral treatments [17], adherence to yoga and exercise interventions in this clinical trial was significantly correlated with baseline variables including depression, fatigue, and physical aspects of quality of life. Demographic variables were not reliable predictors of adherence. It has been previously noted that factors that can be changed, such as mood and social support, more strongly influence adherence than factors that cannot, such as age and gender [18]. While adherence in the present study was relatively low compared to what might be expected in a drug trial, adherence has been observed to be generally lower in behavioral interventions and also with more objective adherence measures compared to self-report [18]. For this reason, we relied on objective class attendance as our primary adherence measure although the two self-reported measures were highly correlated with the objective measure.

The underlying mechanisms associated with greater adherence are not well understood. Because lower adherence, even in the placebo arm of a double blind trial, is associated with worse outcomes including greater mortality [2-4], it has been postulated that those with greater adherence may engage in many other health promoting behaviors. Thus, adherence may be a marker for a personality or related trait related to motivation or goal-directed behaviors. Self-efficacy, which may relate to motivation, is the perceived confidence in one's ability to accomplish a specific task [19]. While self-efficacy was not assessed in this study, it has been shown to be an important correlate of adherence [20]. Within the conceptual framework of self-efficacy, adherence is promoted by the belief that an intervention will be effective (the outcome expectancy) as well as the belief that the individual is capable of following the requirements of the intervention (the efficacy expectancy). When these expectations of success contribute to high self-efficacy, high adherence can result, and moreover, there is reciprocity; subjects who are highly adherent to an intervention may be strengthening their outcome and efficacy expectancies [21]. Expectancy of outcome, besides contributing to adherence, is also a major component of the placebo effect [22,23]. Thus, investigations of factors that predict adherence (or, for that matter, all clinical investigations) are likely to benefit from a good measure of expectancy, which was absent in the present study. The relationship between adherence and health outcomes may be due to mechanisms underlying mind-body interactions, which makes this an area of special interest for researchers conducting mind-body interventions.

Many complementary treatments take a patient-centered approach and utilize outcome and efficacy expectancies, but also require greater effort than simple pill-taking, and this unique set of qualities recommends controlling for factors that impact adherence in mind-body interventions. A discussion of adherence to Mindfulness-Based Stress Reduction programs [24] has identified elements of the intervention itself such as active participation, personal follow-up, accommodation of individual preferences, and emphasis on process instead of outcome, which are believed to effectively discourage attrition and relapse. These are common elements to other mind-body therapies, including both physically active interventions in the present study, which may contribute to differences with conventional clinical trials in adherence and in outcomes.

Improving adherence in a mind-body intervention has the potential to enhance the treatment effect, by increasing the dose of the intervention received by all subjects, especially those who (because of lower baseline functioning) might sustain the greatest benefit and are at greater risk for low adherence. These same subjects who may be most in need of intervention may fail to even meet inclusion criteria for some clinical trials where high adherence during a screen-in period is required. In these cases, results of the intervention can be difficult to generalize and potential magnitude of the treatment effect may be obscured by the relatively high baseline health status of subjects who do pass the adherence run-in phase [3]. In the present study, subjects with a profile of self-reported baseline scores that was low on physical functioning and general health and high on fatigue were more likely to drop out, and less likely to maintain adherence if they did complete the study. This pattern of results accords with other published reports of factors that predict adherence to treatment, implying that at least some commonly identified determinants are equally relevant for mind-body interventions.

Reviews of efforts to improve adherence to therapeutic regimens have thus far been inconclusive, and suggest that no single approach is consistently effective for all subjects in all interventions [25,26]. Strategies to promote adherence include making instructions to subjects simpler and less demanding, addressing cognitive-motivational factors such as self-efficacy and health beliefs, offering social support and reinforcement, and providing reminders, with results suggesting that highest success rates are achieved by a combination of such approaches. Specific strategies may be particularly important for groups of subjects that may have more difficulty with adherence to behavioral interventions because of a variety of issues such as getting to a class, physical limitations, parental or caregiver responsibilities, or, as already discussed, depression. Concerns such as these may be especially relevant for elderly populations [27], like those tested in the present study, who stand to gain greatly from improvements in health. Continued attention to maximizing adherence is important for enhancing treatment benefits, as well as controlling the costs of clinical trials and increasing statistical power to determine the effectiveness of interventions [28].

This analysis examined study completion or attrition as well as adherence to the terms of a physically active intervention, and it may be that unique factors differentiate correlates of these related measures. As previously mentioned, lower adherence, even in the placebo arm of a double blind trial, is associated with worse outcomes [2-4], although the underlying mechanisms linking adherence to health outcomes are still unknown. Future trials may elucidate this relationship, which could be related to mind-body interactions.

## Conclusion

Healthy seniors have reasonably good attendance at classes with a physically active intervention. There were no clear effects of adherence on the significant study outcomes (quality of life and physical measures). Adherence to the intervention was significantly correlated with baseline measures of depression, fatigue, and physical components of health-related quality of life. Adherence was not related to age, gender, or education level. Decreased adherence to a potentially beneficial intervention for fatigue or depression has the potential to decrease the effect of the intervention in a clinical trial because subjects who might sustain the greatest benefit will receive a lower dose of the intervention and subjects with higher adherence rates may be functioning better before the intervention. Strategies to maximize adherence among subjects at greater risk for low adherence will be important for future trials, especially complementary treatments requiring greater effort than simple pill-taking.

## Competing interests

The author(s) declare that they have no competing interests.

## Authors' contributions

KEF performed data analysis and drafted the manuscript. SS supervised subject recruitment and interventions, and obtained subject data. DZ supervised data management and obtained subject data. MH helped with overall design and execution of study. BSO conceived of the study, participated in its design and coordination. All authors read and approved the final manuscript.

## Pre-publication history

The pre-publication history for this paper can be accessed here:


